# Movement Prototypes in a Complex Teamgym Gymnastics Technique on the Vaulting Table and Their Relationship with Judges’ Scores

**DOI:** 10.3390/s23063240

**Published:** 2023-03-18

**Authors:** Joana Barreto, Rui Henriques, Sílvia Cabral, Bruno Pedro, César Peixoto, António Veloso

**Affiliations:** 1 CIDEFES, Universidade Lusófona, 1749-024 Lisbon, Portugal; 2 INESC-ID, IST, Universidade de Lisboa, 1000-029 Lisbon, Portugal; 3 Laboratório de Biomecânica e Morfologia Funcional, Faculdade de Motricidade Humana, CIPER, Universidade de Lisboa, 1495-751 Cruz Quebrada Dafundo, Portugal; 4 Laboratório de Perícia no Desporto, Faculdade de Motricidade Humana, CIPER, Universidade de Lisboa, 1495-751 Cruz Quebrada Dafundo, Portugal

**Keywords:** kinematic analysis, inertial sensors, cluster analysis, execution scores, optimal movement variability, performance, sports, Teamgym Code of Points, Xsens MVN Link

## Abstract

A successful high-level gymnastics performance is the result of the coordination and inter-relation of body segments to produce movement prototypes. In this context, the exploration of different movement prototypes, as well as their relations with judges’ scores, can aid coaches to design better learning and practice methodologies. Therefore, we investigate if there are different movement prototypes of the technique of the handspring tucked somersault with a half twist (HTB) on a mini trampoline with a vaulting table and its relations with judges’ scores. We assessed flexion/extension angles of five joints during fifty trials, using an inertial measurement unit system. All trials were scored by international judges for execution. A multivariate time series cluster analysis was performed to identify movement prototypes and their differential association with judges’ scores was statistically assessed. Nine different movement prototypes were identified for the HTB technique, with two of them associated with higher scores. Statistically strong associations were found between scores and movement phases one (i.e., from the last step on the carpet to the initial contact of both feet with the mini trampoline), two (i.e., from the initial contact to the take-off on the mini trampoline) and four (i.e., from the initial contact of both hands with the vaulting table to take-off on the vaulting table) and moderate associations with movement phase six (i.e., from the tucked body position to landing with both feet on the landing mat). Our findings suggest (a) the presence of multiple movement prototypes yielding successful scoring and (b) the moderate-to-strong association of movement variations along phases one, two, four and six with judges’ scores. We suggest and provide guidelines for coaches to encourage movement variability that can lead their gymnasts to functionally adapt their performance and succeed when facing different constraints.

## 1. Introduction

As gymnastics skills are performed in a relatively stable and predictable environment, they are generally categorised as closed skills [1]. For this reason and until today, coaches follow methodologies that promote automaticity of the movement and reduce its variability [2]. An example is the use of a measuring tape along the approach run area for vaulting, assuming that gymnasts will perform a stereotyped approach run [3,4] with similar biomechanical parameters (e.g., step length, velocity, vertical impulse, etc.) in every trial. Still, it is not uncommon to observe gymnasts pulling out of the attempt during the approach run, finding it unfamiliar or suffering accidents such as hitting the vaulting table during training and competition [4].

More recent studies grounded in the Ecological Dynamics perspective [5,6,7,8,9] have demonstrated that variability in performance is present and depends on the individual (e.g., fatigue, emotions and intentions), environmental (e.g., championships final, qualifications and training) and task (e.g., verbal instructions and time) constraints [6,7,8]. Inter-variability (i.e., the variation in movement parameters between gymnasts when performing the same task [10,11]) was observed during tasks such as the handstand and handspring front somersault on vaulting [12,13]. Intra-variability (i.e., the variations in movement parameters along repetitions of the same task performed by the same subject [10,11]) was observed for step velocity [14] and length, duration of the approach run [15], last step–springboard distance [16], hands–edge of the vaulting table distance, body position during vaulting table take-off [3] and duration of contact to the vaulting table [14]. Together, these results reinforce the hypothesis of a functional movement regulation.

**Figure 1:**
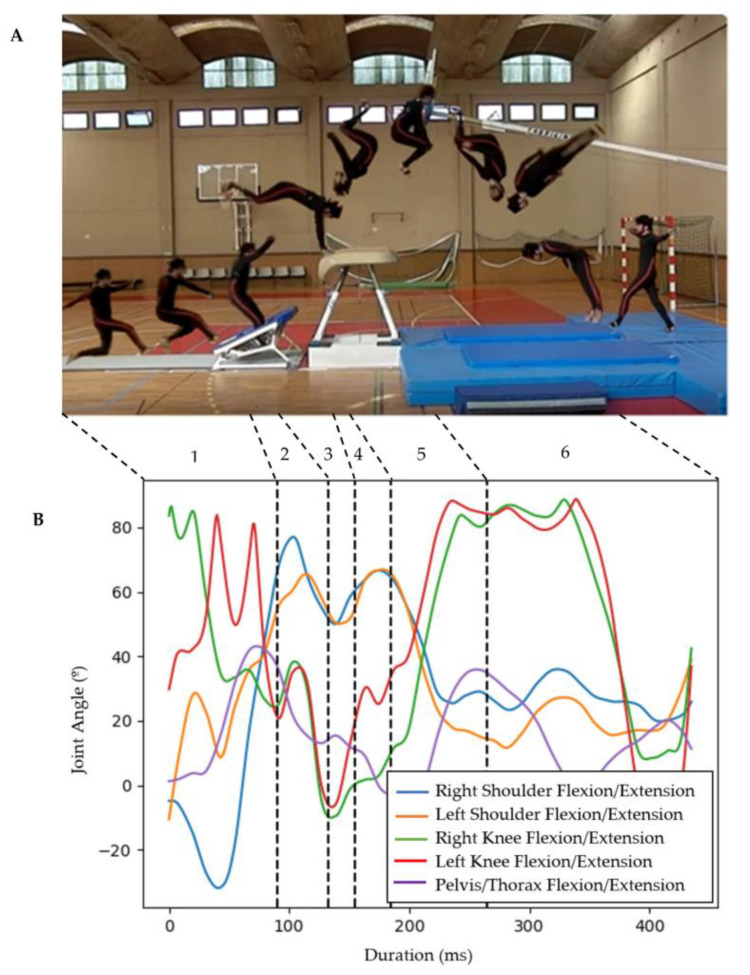
(**A**) After a maximum 25 m approach run, the gymnast performed a take-off from the mini trampoline followed by a forward entrance placing both hands on the vaulting table (support phase), one and a half somersault in a tucked position (total of 720° on a transversal axis rotation) and a half twist (180° on a longitudinal axis rotation) before landing. The dotted lines and numbers represent the movement phases analysed: (1) from the last step on the carpet to the initial contact of both feet with the mini trampoline (mean duration = 0.39 s ± 0.03); (2) from the initial contact to the take-off on the mini trampoline (mean duration = 0.17 s ± 0.02); (3) from the take-off on the mini trampoline to the initial contact of both hands with the vaulting table (mean duration = 0.06 s ± 0.03); (4) from the initial contact of both hands with the vaulting table to take-off on the vaulting table (mean duration = 0.15 s ± 0.04); (5) from the take-off on the vaulting table to achieving the tucked body position during the aerial phase (mean duration = 0.30 s ± 0.04); and (6) from the tucked body position to landing with both feet on the landing mat (mean duration = 0.75 s ± 0.11). (**B**) Joint angle variation along the 6 different phases of movement of one trial of the HTB. The dotted lines illustrate the beginning and the end of the phases of movement.

In gymnastics disciplines such as Teamgym, the Code of Points [17] allows some variability of movement without deductions in the execution score, namely, for joint angle amplitude. Examples are the body position during the take-off from the vaulting table (Figure 1A, at the end of 4), where knees, hips, shoulders and elbow angles must be >135°, and the body position during landing (Figure 1A, at the end of 6), where a ≤90° flexion of the knees and hips are allowed.

Some limitations in the existing literature prompted this study. First, the tasks analysed in previous works are relatively simple (e.g., handspring on vaulting table) due to the challenges in collecting and analysing biomechanical data outside the laboratory. These tasks are generally performed in artistic gymnastics, where a springboard is used instead of a vaulting table as happens in Teamgym, which can lead to different biomechanical behaviours. Second, and despite the growth of Teamgym in Europe and other countries, few scientific studies have been conducted for Teamgym, and they are mostly related to injuries in the sport. Finally, most of the studies in gymnastics biomechanics analysed single data points of individual variables without considering the interaction and synergies of body components.

To address these limitations, we made a multivariate time series cluster analysis to identify movement prototypes [18] of a complex task performed in Teamgym. We also investigated the variation in the knees, shoulders and pelvis/thorax flexion/extension angles of each movement prototype, considering the interaction of body components during complex movements [19]. We benefitted from the recent advances in inertial measurement unit systems to collect and analyse biomechanical data during the performance of a complex skill in an ecological environment.

Considering the mentioned studies, we aimed to (a) investigate if there are different movement prototypes of the technique of the handspring tucked somersault with a half twist (HTB) on a mini trampoline with a vaulting table, resulting from a functional regulation of movement and consequently showing intra- and inter-movement variability, and (b) assess the correlation between those movement prototypes with judges’ scores. Movement prototypes were explored for the whole task duration as well as for relevant phases of the task with the complementary aim of studying phase-specific variations and identifying which phases of movement are more related with scores.

We hypothesized that (1) different movement prototypes are identified because of a functional movement regulation and intra- and inter-variability of movement, and (2) different prototypes could show differing scores, demonstrating that not only a functional movement regulation strategy is present and is acceptable from judges, but that coaches need to adapt practice design. The fifth and sixth movement phases (i.e., from the take-off on the vaulting table to landing with both feet on the landing mat) are commonly seen as the most important phases of movement, especially for two reasons: first, it is when the majority and most important motor actions occur, and second, it is when judges apply more execution deductions according to the Teamgym Code of Points [17] (e.g., body position when leaving the vaulting table, body shape during the aerial phase, height, twisting position and timing, control during landing, etc.). Despite that, we did not consider any conditioning hypothesis for these movement phases due to the influence of previous phases, namely, the approach run.

## 2. Materials and Methods

### 2.1. Participants

Altogether, 13 elite Teamgym gymnasts (n = 13; mean age: 25.2 ± 2.83 years; mean experience = 10.8 years) with international experience participated in the study. All gymnasts were able to perform safely and successfully the proposed skill. Five international Teamgym judges from Portugal, Italy and the Czech Republic scored execution according to the Teamgym Code of Points [17]. The study was conducted in accordance with the Declaration of Helsinki and approved by the Faculty Ethics Committee (1/2020; 24 January 2020). Gymnasts signed an informed consent form prior to starting the experimental procedures and declared that they were free from injuries and able to perform the tasks autonomously.

### 2.2. Task

Participants performed a handspring tucked somersault with a half twist (HTB) on a mini trampoline with a vaulting table (MTVT) (Figure 1).

### 2.3. Instruments and Procedure

The data were collected in a sports hall using a carpet that was 25 m in length, 2 m in width and 3.5 cm thick, a mini trampoline, a vaulting table and a landing area according to the official Teamgym directives [20]. We recorded biomechanical data using a system of seventeen inertial measurement sensors (Xsens MVN Link; Xsens Technologies, Enschede, The Netherlands) at a rate of 240 Hz. This system was previously validated against an optoelectronic system to measure kinematic variables during the performance of gymnastics tasks in an ecological environment [21]. The study revealed a very good to excellent coefficient of multiple correlation values, as well as acceptable errors (root mean square errors) for the joint angles considered in the present study. The IMUS Xsens MVN Link consists of five MTx sensors (placed on the pelvis, sternum, hands and head) and twelve MTx-STR sensors (placed on the legs and upper body). Both inertial unit sensors contain 3D linear accelerometers to measure accelerations (as gravitational acceleration), 3D rate gyroscopes to measure angular velocities, 3D magnetometers to measure earth magnetic field and a barometer to measure atmospheric pressure. The placement of sensors and calibration conformed to the manufacturer’s recommendations. Kinematic data were processed in multilevel high definition using MVN Analyse software (Version 2019.2.1, Xsens Technologies, Enschede, The Netherlands). We used acceleration peaks representing foot and hand impacts [22] on the carpet, mini trampoline, vaulting table and landing mat, to define six phases of movement: (1) from the last step on the carpet to the initial contact of both feet with the mini trampoline; (2) from the initial contact to the take-off on the mini trampoline; (3) from the take-off on the mini trampoline to the initial contact of both hands with the vaulting table; (4) from the initial contact of both hands with the vaulting table to take-off on the vaulting table; (5) from the take-off on the vaulting table to achieving the tucked body position during the aerial phase; and (6) from the tucked body position to landing with both feet on the landing mat (Figure 1). We calculated segment positions and orientations using MVN Fusion Engine for Xsens and considered for analysis the time series for Flexion/Extension angles (°) of the left and right shoulders, left and right knees and pelvis/thorax.

All trials were recorded using a video camera (Casio EXILIM EX-F1, 60 Hz, Casio Computer Co., Ltd., Tokyo, Japan) placed perpendicular to the MTVT. Participants performed a twenty-minute warm-up and several practice trials before instrument calibration was performed. Participants were informed that trials would be evaluated by international Teamgym judges and performed five trials as if they were in a competition. Participants chose the distance of the run-up and instrument calibrations were repeated before each trial. Each participant was present in one session and performed five trials of the HTB. They were allowed to rest for some seconds between trials. Following the completion of all trials, participants were fully debriefed.

### 2.4. Data Analysis

#### 2.4.1. Statistical Procedures

A total of fifty trials were considered for analysis (mean trials per participant = 3.85 ± 0.55), with the remaining fifteen trials excluded due to falls after landing on the mat, not performing the requested movement (e.g., performing a straight instead of a tucked body position during the aerial phase) or due to poor quality of data. We defined an individual movement as a 5-order multivariate time series, monitoring the variation in the 5 measured body angles along the whole HTB task for each phase.

Given a set of trials, a subset of individual movements that satisfy specific similarity criteria among each other and dissimilarity criteria against remaining movements was considered to identify movement prototypes. To this end, multivariate time series clustering analysis was first performed to partition individual movements onto a set of cohesive and well-separated groups (clusters), which were subsequently considered to retrieve the movement prototypes via time series averaging [23].

Dynamic time warping (DTW) distance was considered to rigorously assess pairwise similarity between multivariate signals [24,25]. An illustrative instantiation of DTW elastic behaviour over two individual movements for the target task is presented in Figure S1 (Supplementary Materials). DTW is selected over alternative distances for three major reasons. First, DTW was originally designed for the analysis of movement signals [24,25]. In particular, its elastic nature is essential to tolerate/accommodate temporal misalignments between individual movements, being able to adequately handle the natural intra-personal and inter-personal variability. Second, DTW is well-prepared to simultaneously account for the inherent temporal and multivariate dependencies observed of the target angle signals. Finally, the elastic properties of DTW are able to assess similarity between signals with arbitrarily high different durations, as in the targeted case.

Time series k-Means [26,27] using DTW distance was considered to produce a clustering solution for the whole and phase-specific HTB. In contrast with classical k-Means, the computation of centroids was not produced under the traditional multivariate averaging principle but considering DTW averaging. In this context, the centroid of a cluster of individual movements is an expressive multivariate signal that is not subjected to aliasing effects caused by temporal misalignments. DTW barycentre analysis was applied not only to compute the centroids along the clustering task, but also to extract the movement prototypes from the final clustering solution, here termed movement prototypes.

#### 2.4.2. Statistical Validation

To identify the optimal number of movement prototypes for the whole HTB and each phase, the number of clusters were varied between 2 (minimum) and 15 (placed upper bound to guarantee a good representativity of movements per cluster) and two major methods were considered. First, elbow analysis of the sum of squared errors. Second, the optimization of the silhouette along the eligible elbow points.

The learnt clustering solutions under the optimal number of movement prototypes were further subjected to a strict quality assessment by statistically testing the observed silhouette against null data models (angle permutations within and across individuals) to ensure the cohesion and separation of the clustered movements.

Finally, an association study of the clustered movements (for the whole HTB and each phase) against judges’ scores was undertaken. To this end, two major groups of statistical tests were considered. First, to test whether the set of movement prototypes produced along the HTB task (or specific phase) show differential scores, ANOVA was applied under a significance threshold (alpha = 0.01) to assess generalized statistically significant scoring differences across the given prototypes. ANOVA F-testing was applied with K-1 (between-group), N-K (within-group) and N-1 (total) degrees of freedom, where K is the number of clusters for the HTB task (or specific phase) and N is the number of trials. Second, as ANOVA testing may not ensure that differential scores are observed for every pair of movement prototypes from the learnt multi-wise set of patterns, we further conducted a pairwise statistical assessment of score differences. To this end, *t*-test with independent samples was applied considering the scored movements for each pair of clusters when scores passed the Shapiro–Wilko normality test. Otherwise, the corresponding non-parametric Wilcoxon signed-rank testing was considered.

## 3. Results

### 3.1. Handspring Tucked Somersault with a Half Twist—Global Analysis

**Figure 2:**
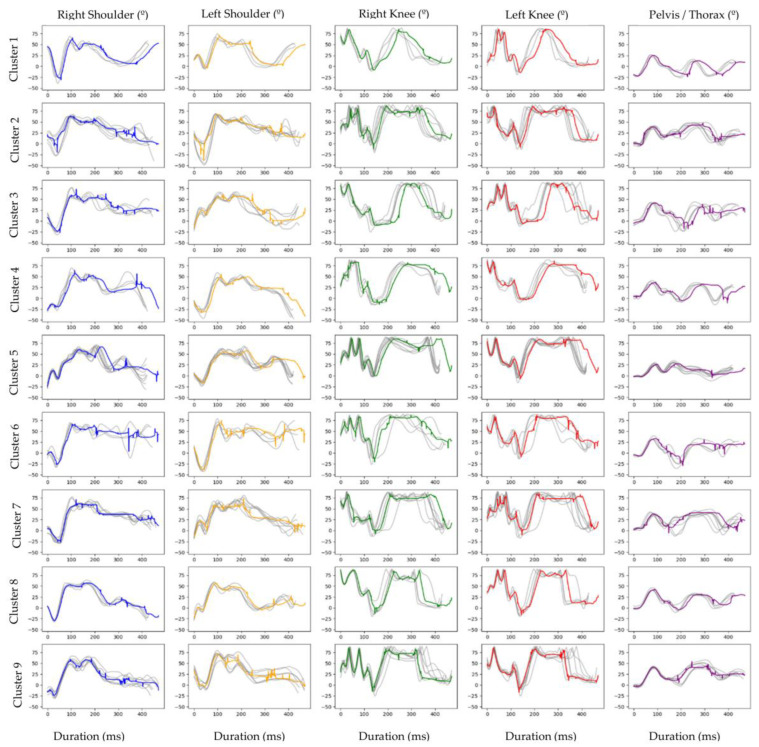
Cluster times series for the joint angles of the complete HTB. In each cluster, the grey lines represent the time series of each trial. The coloured lines represent the movement prototype that is the result of the average trials of each cluster and each joint angle.

Nine clusters characterised the performances of the complete HTB (Figure 2). Each cluster comprises similar temporal series trials, considering the five joint angles in the analysis. In addition, each cluster includes a movement prototype as the result of the averaged trials. Different clusters represent significant different temporal series of joint angles, meaning different motor behaviours during the performance of the HTB.

Clusters two, seven and nine include seven trials (14.29%), clusters four, five and eight include four trials (8.16%), cluster three involves six trials (10.2%), while clusters one and five include three (6.12%) and eight (16.33%) trials, respectively. The quality assessment of the undertaken clustering analyses for the different movement prototypes is included in the Supplementary Materials (Figures S2 and S3).

**Figure 3:**
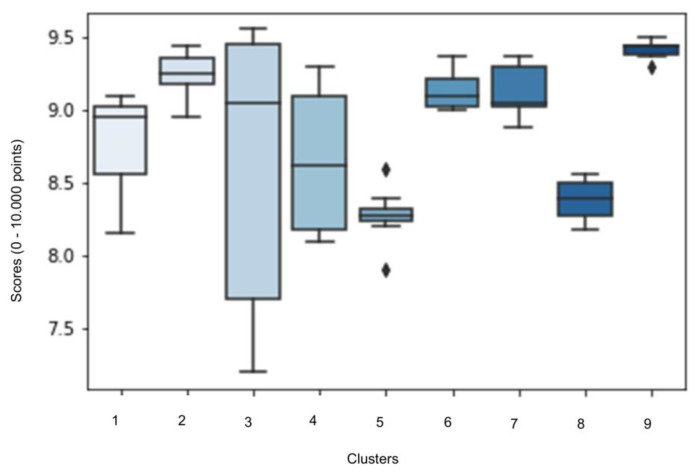
Boxplots with average and standard deviation scores for each one of the nine clusters that characterise the performance of the HTB. F = 5.859.

Cluster nine has statistically significant higher scores compared to the remaining clusters (cluster one, *p* = 0.017; cluster two, *p* = 0.038; cluster four, *p* = 0.012; cluster five, *p* = 0.0003; cluster six, *p* = 0.024; cluster seven, *p* = 0.004; and cluster eight, *p* = 0.006), except when compared to cluster three (*p* = 0.530) (Figure 3). Based on the average time series or movement prototype of cluster nine, the HSB is characterised by a strong shoulder flexion (70°) and pelvis–thorax flexion (45°) during the first phase; a slight flexion (20°) and the initiation of an extension (40°) of the knee, together with an extension of the shoulder and pelvis–thorax (20°) during the second phase; a maintenance of the angular positions of the shoulders, knees and pelvis–thorax (≤10°) during the third and fourth phases, except an accentuated knees flexion during the fourth phase (40°) to prepare for the tucked body position; the continuation of the knees (40°) and pelvis–thorax flexion (30°) during the fifth phase, while a shoulder extension is observed (50°) in order to rotate in a tucked body position (hands on the knees); and finally, a knee and pelvis–thorax extension (40°) to a straight body position followed by a flexion (20°) during the initial contact with the landing mat, while the shoulders demonstrate a minor flexion (15°), finishing with an extension (25°) to smooth the landing.

### 3.2. Handspring Tucked Somersault with a Half Twist—Phases Analysis

**Figure 4:**
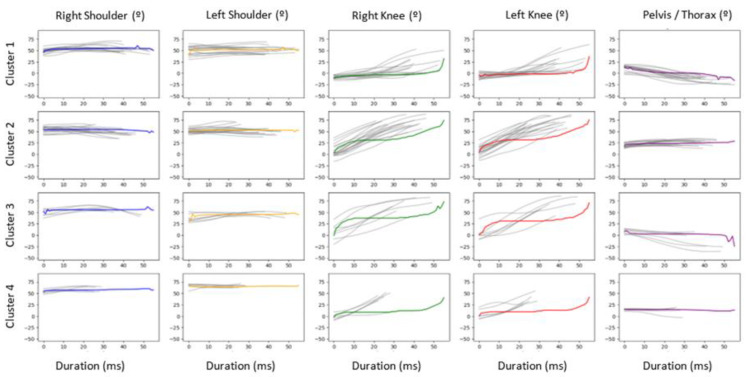
Example of four cluster times series for phase 4 (i.e., from take-in to take-off on the vaulting table). In each cluster, the grey lines represent the time series of each trial for this phase of movement. The coloured lines represent the movement prototype that is the result of the average trials of each cluster and each joint angle.

We considered six phases of movement (Figure 1). In all movement phases, each cluster comprises trials with similar temporal series (grey lines, Figure 4), considering the five joint angles in the analysis. Additionally, every cluster includes a movement prototype that is the result of the averaged trials (coloured lines, Figure 4). Different clusters represent trials with significant different temporal series of joint angles, meaning different motor behaviours during the different phases of the performance of the HTB.

Phase one includes seven clusters, phases two and five comprise three clusters, and finally phases three, four (Figure 4) and six contain four clusters.

**Figure 5:**
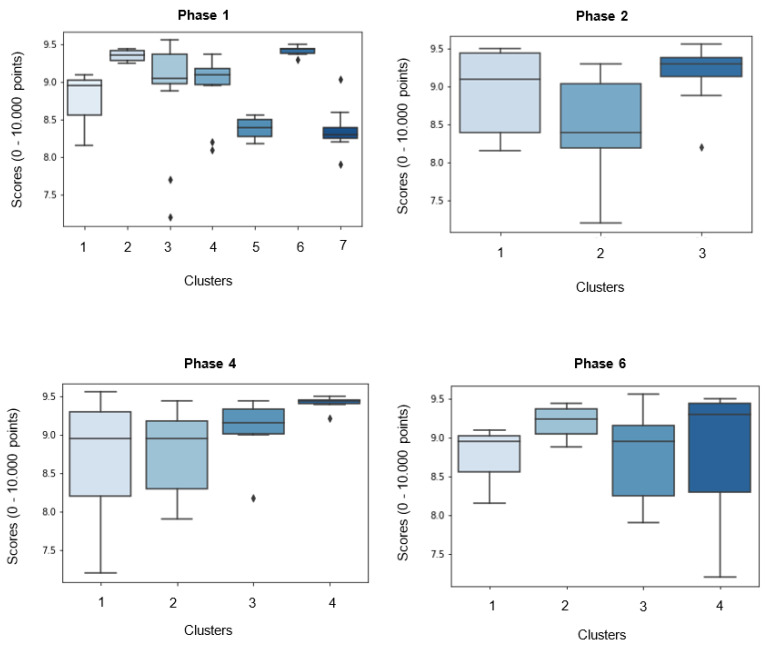
Boxplots with average and standard deviation scores for each cluster of the phases of movement that have strong (1st, 2nd and 4th phases of movement) and moderate (6th phase of movement) associations with scores.

Statistically strong associations were found between scores and phases one (*F* = 5.083, *p* = 0.001), two (*F* = 12.303, *p* < 0.001) and four (*F* = 3.192, *p* < 0.032) and moderate associations with phase six (*F* = 2.074, *p* < 0.117) (Figure 5). This result reflects that in each of these movement phases there are statistically significant different scores for different clusters. During the first phase, clusters two and six show the highest average scores and a small standard deviation (SD). During the second phase, cluster three has a higher average score than the remaining clusters. Lastly, cluster four registered the highest average scores for phases four and six.

## 4. Discussion

This study aimed to (a) investigate if there are different movement prototypes of the technique of the handspring tucked somersault with a half twist (HTB) on a mini trampoline with a vaulting table, and (b) assess the correlation between those movement prototypes with judges’ scores. A functional movement regulation strategy was confirmed with the identification of nine different movement prototypes for the technique in this study, with intra- and inter-variability arising during performance. This result demonstrates that, even with elite samples, the same task can be successfully performed with different movement prototypes. Additionally, and as expected, different scores were given by judges for each prototype, with prototypes three and nine achieving the highest and statistically similar scores.

The current study showed that the trials from eleven gymnasts were assigned constantly to the same clusters, demonstrating consistency of movement over various trials. This consistency of movement may reflect their elite level, although we observed some variability of joint angles between the trials in each cluster (Figure 2). This movement variability might reflect what is named as metastable attunement [28] meaning that gymnasts used the learned patterns of behaviour to perform the task while being sensitive to the present constraints (e.g., individual, environmental and task constraints), adapting their movement to successfully perform. Being a technical sport, where judges are trained to find imperfections in movement, a low variability of movement should be desirable in critical aspects of the technique, while less relevant aspects that are not so determinant for the score can experience more variability [12,29] as the result of a functional interaction with constraints [30,31,32,33]. Therefore, more important than avoiding movement variability, the success resides in keeping the critical biomechanical parameters consistent (but not rigid) and adjusting the less critical aspects of movement during specific movement phases. The results depicted demonstrate the existence of different movement prototypes to perform the HTB and movement variability between trials in the same cluster. This means that, from a stable performance, gymnasts adapted their movement in a functional and optimal manner, maintaining the balance between movement stability and flexibility [28,32]. This metastable attunement [28] allowed gymnasts to achieve the main goal of the HTB, independently of their scores. We can question if they would be able to achieve the main goal of the task if they performed a stereotyped and rigid stable movement pattern. It can also be possible that this metastable attunement enables the gymnast to adjust their motor behaviour to avoid a less successful landing on the mat or even an injury, which would not be possible on the basis of performing repetitively a stereotyped and automatic movement pattern [32].

Two gymnasts had their trials assigned to two different clusters with a statistically significant impact on scores. This might indicate that, although some joint amplitude variability in specific phases of movement does not reverberate on the score, an increase in variability beyond an optimal level [33] may result in technical incorrections (e.g., joint amplitudes over the limits defined in the Code of Points), leading to additional deductions in scores.

From the nine clusters identified during the performance of the HTB, the third and ninth are associated with significantly higher scores, despite cluster three displaying a considerable standard deviation for score (the lower scores resulted from corrections to gain control after landing to avoid falling and consequently a higher score deduction). First, this result may support the possibility that an optimal level of movement variability still leads to greater scores (≥9.000 in 10.000 possible points), and second, corrections to gain control of landing (i.e., multiple steps, a large rebound jump, etc.) do not significantly reduce scores. Considering these findings, we encourage coaches and gymnasts to use this acceptable movement variability to their advantage, meaning that instead of instigating a perfectly controlled landing (i.e., perform landing without any additional step or rebound jump), a more fluid, yet controlled, movement can be used as a strategy to progressively dissipate the body energy and reduce the impact of landing on the lower joints.

To better understand which movement phases yield strong associations with scores, we performed phase-conditional clustering analyses, followed by a score-aware analysis of variances. The numbers of clusters allocated to the six phases of movement show that a diversification of movement prototypes is more likely to occur along some phases, which is moderately associated with movement variability. Results showed that statistically significant differences for scores occurred for movement during phases one, two, four and six, confirming the significant correlations of these phases with judges’ scores [4,34,35,36], while phases three and five have less influence on scores, showing a similar range of scores for all clusters.

Since gymnasts that participated in this study are right and left-handed, this may have contributed to (without totally explaining it) a superior number of clusters in the 1st phase of movement. During this phase gymnasts start to approach an optimal body position to contact the mini trampoline, so the 1st flight/3rd phase (i.e., from mini trampoline take-off to the initial contact with vaulting table) will be a lower parabola with a minimal loss of velocity and greater energy and velocity [37]. This optimal body position is conditioned by the approach run characteristics as velocity [38,39], individual characteristics, namely, height [40], strength [40], power, flexibility, biological development, visual perception and strategy [4,15,41] and other constraints, which in practice may reveal a larger variability and adjustment of movement, consequently resulting in the higher number of clusters observed. Figure 5 demonstrates that four clusters (two, three, four and six) with different movement prototypes for phase one have very good average scores (≥9.000 points), reinforcing the acceptance of an optimal and needed movement variability, without considerably reducing score.

The second and fourth phases present an inferior number of movement prototypes (Figure 5), demonstrating that these phases have a limited range of movement variability, yet it is present. We observe a significant lower score for cluster three on second phase and for clusters one and two during the fourth phase, confirming that, at least for the variables considered, the range for variability of movement prototypes are reduced. During both phases, gymnasts body position [42], specially hip flexion/extension angles [36], velocity and force applied to mini trampoline and vaulting table are crucial for success [36,39], which corroborates the strong associations with score that we found. Despite the short duration of hands contact with vaulting table, it is possible that elite gymnasts make small modifications in linear and angular momentum to improve the distance, height and body rotation during the second flight [43] (phases five and six, between vaulting table take-off and landing). Again, and despite the restricted limits of joint flexion/extension variability during these phases, assume a functional adaptation of behaviour to make the most of their contact with mini trampoline and vaulting table. This functional and essential movement variability, resulting from the interaction between gymnasts and several constraints, can lead to a better performance of the second flight (phases five and six) and consequently to higher scores [17]. On phase 2, trials from cluster 3 demonstrated less variability for joint angles and a higher average score (with a small SD), while during the phase four, clusters with higher scores (three and four) had a significant variability in joint angles (differences between 35° and 20°, respectively), leading us to suggest, that (1) during contact with mini trampoline (phase two), a lower variability on joint angles and movement sequence is desirable for a higher score; and (2) during contact with vaulting table (phase four), some variability on joint angles amplitudes does not negatively influences scores. This may indicate that, as we mentioned before, the second phase of movement may include more critical biomechanical parameters comparing to the fourth phase of movement, demanding a reduced movement variability.

The sixth and last phase of movement, from a tucked body position to the initial contact with landing mat, has a moderate association with scores (Figure 5). This phase, together with phase five, is facilitated by a high velocity during the approach run [36,38] and a great kinetic energy created during the contact with vaulting table [44,45], remembering that even the last movement phases are a consequence of the previous phases. The results reveal that, cluster four has a higher average score but presents a great variability for joints amplitudes, resulting in a significant standard deviation for scores. Although this cluster represents the most correct movement prototype and sequence, some joints amplitudes lead to more deductions and consequently lower scores. This result would be expected since the Code of Points [17] directs judges to especially focus (and apply deductions) on performance of phases five and six (i.e., the aerial phase), where it is expected that judges are less tolerant to the variations of joint amplitudes. Contrary, less movement variability on cluster one did not cause higher scores, due to a less correct movement prototype (e.g., a late opening from a tucked to a straight body position, a straight body position not well-defined during landing). It is worth to highlight that the aerial phase is strongly influenced by previous movement phases and due to its technical complexity, time to perform and other constraints, it might be difficult to gymnasts to adjust biomechanical parameters and correct or improve their performance. Our findings seem to depict that during the last phase of performance of HTB, an optimal movement variability can be accepted by judges (e.g., according to the Code of Points [17], judges should consider a tucked body position when the hips and knees joint angles are equal or less than 135°, instead of having to demonstrate a precise joint angle), provided that the gymnasts perform with technical correction (e.g., correct timing to initiate and complete longitudinal body axis rotation, to show an extended body position before landing, among others).

## 5. Conclusions

The major implication of this study is that, even when considering an elite sample, different movement prototypes arise when performing an HTB, with two of them showing statistically significant higher scores against alternative prototypes. This led us to conclude that an optimal amount of movement variability is beneficial for gymnasts’ performances. These results point to the need to design representative learning/practice tasks that promote a relationship and interaction between gymnasts and key performance constraints, so gymnasts can eventually explore and adjust their behaviours, based on their individual characteristics and be more prepared to face the changing constraints with success [2,7,8,46].

Movement prototypes along the first, second, fourth and sixth phases of the HTB movement show the strong and moderate association with judges’ scores, emphasizing their importance. However, an optimal movement variability, comprising a balance between stability and flexibility, did not negatively influence scores, despite it being more or less tolerable by judges depending on the movement phase.

From a pedagogical and applied perspective, based on the results of this study, we would like to make some suggestions to coaches. First, intra- and inter-variability that occur during the learning processes and technical performance should be embraced, recognizing the individual characteristics of each gymnast, as well as the individual state of readiness. To design an appropriate task [47], more than consider the outcomes, coaches should consider the commitment of the gymnast to the goals and learning process as well as be prepared to evaluate, review and adapt the goals to each gymnast’s characteristics when needed. Finally, the critical components and goals of the task must guide the design of representative learning/practice tasks.

## Data Availability

The data presented in this study are available on request from the corresponding author.

## Supplementary Materials

The following supporting information can be downloaded at: https://www.mdpi.com/article/10.3390/s23063240/s1, Figure S1: Illustrative application of the DTW to two HTB movements from the same gymnast. Each movement is described by a multivariate time series of order 5 corresponding to the angle variation along the 5 monitored joints. The elastic nature of the DTW is able to tolerate temporal misalignments and movements of varying duration; Figure S2: Sum of squared errors (SSE) and silhouette for the different number of clusters for the complete movement. The yellow vertical line represents the best solution for the number of clusters; Figure S3: Sum of squared errors (SSE) and silhouette for the different number of clusters for each phase of movement. The yellow vertical line represents the best solution for the number of clusters; Figure S4: Clusters times series for phase 1 (i.e., from the last step on carpet to the initial contact of both feet with mini-trampoline) considering knees, shoulders and pelvis/thorax flexion/extension angles. The grey lines represent the time series of each trial; Figure S5: Clusters times series for phase 2 (i.e., from the initial contact to the take-off on mini-trampoline) considering knees, shoulders, and pelvis/thorax flexion/extension angles. The grey lines represent the time series of each trial; Figure S6: Clusters times series for phase 3 (i.e., from the take-off on mini-trampoline to the initial contact of both hands with vaulting table) considering knees, shoulders, and pelvis/thorax flexion/extension angles. The grey lines represent the time series of each trial; Figure S7: Clusters times series for phase 5 (i.e., from the take-off on vaulting table to achieving the tucked body position during the aerial phase) considering knees, shoulders, and pelvis/thorax flexion/extension angles. The grey lines represent the time series of each trial; Figure S8: Clusters times series for phase 6 (i.e., from the tucked body position to landing with both feet on landing mat) considering knees, shoulders, and pelvis/thorax flexion/extension angles. The grey lines represent the time series of each trial.
