# Supplementary material for: Movement Prototypes in a Complex Teamgym Gymnastics Technique on the Vaulting Table and Their Relationship with Judges’ Scores
Source: Sensors (Basel). 2023 Mar 18;23(6):3240. doi: 10.3390/s23063240 (PMC10054299; doi:10.3390/s23063240)
Supplement: Supplementary file 1 [file sensors-23-03240-s001.zip › sensors-2146647-SI.pdf]

Supplementary Materials

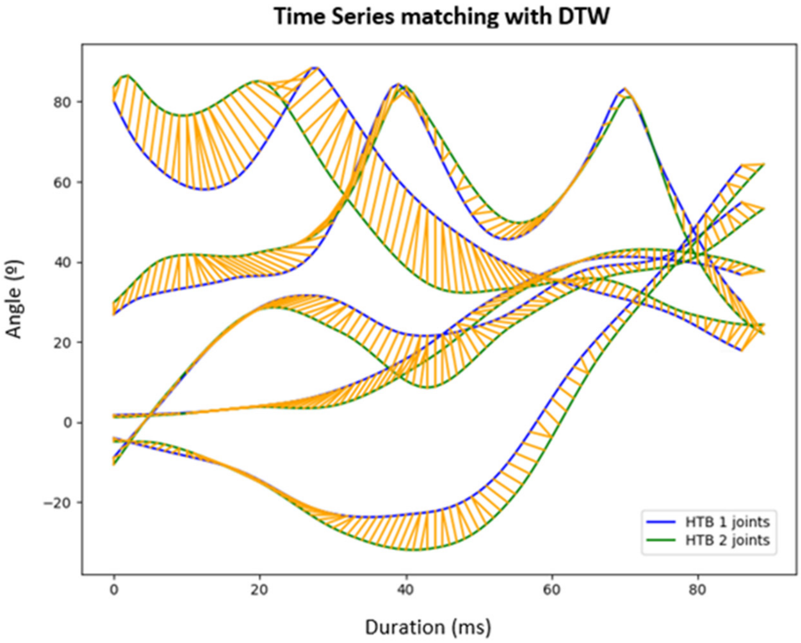

Figure S1. Illustrative application of the DTW to two HTB movements from the same gymnast. Each movement is described by a multivariate time series of order 5 corresponding to the angle variation along the 5 monitored joints. The elastic nature of the DTW is able to tolerate temporal misalignments and movements of varying duration.

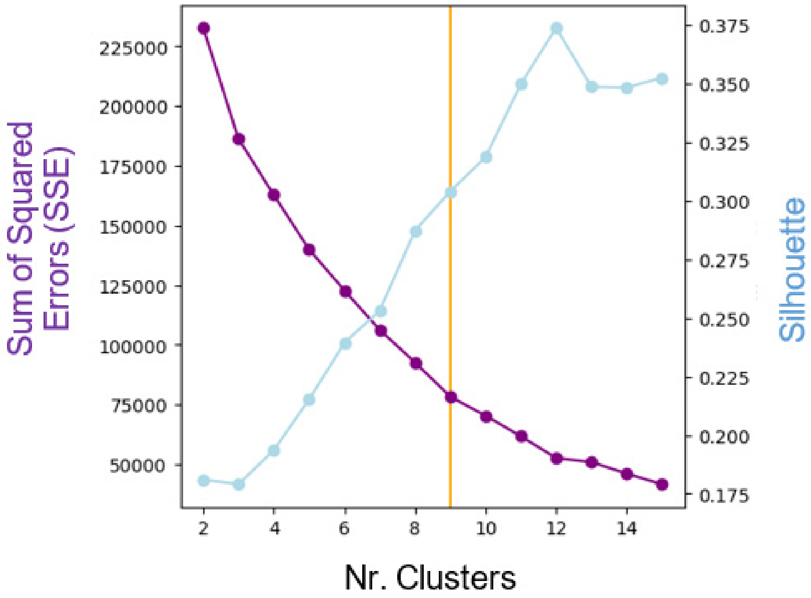

Figure S2. Sum of squared errors (SSE) and silhouette for the different number of clusters for the complete movement. The yellow vertical line represents the best solution for the number of clusters.

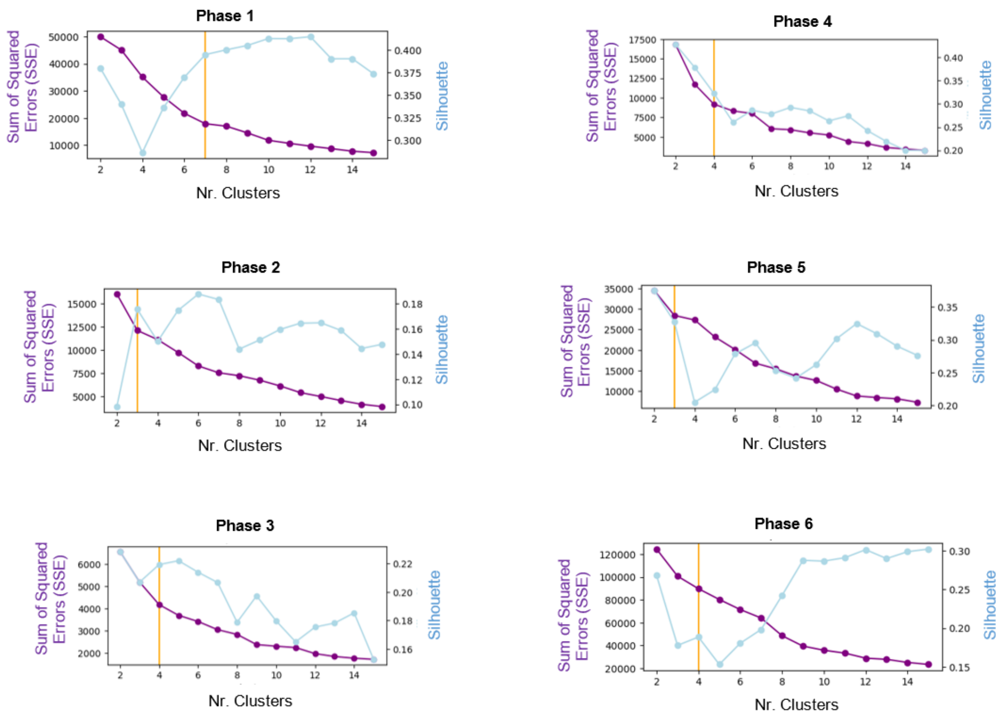

Figure S3. Sum of squared errors (SSE) and silhouette for the different number of clusters for each phase of movement. The yellow vertical line represents the best solution for the number of clusters.

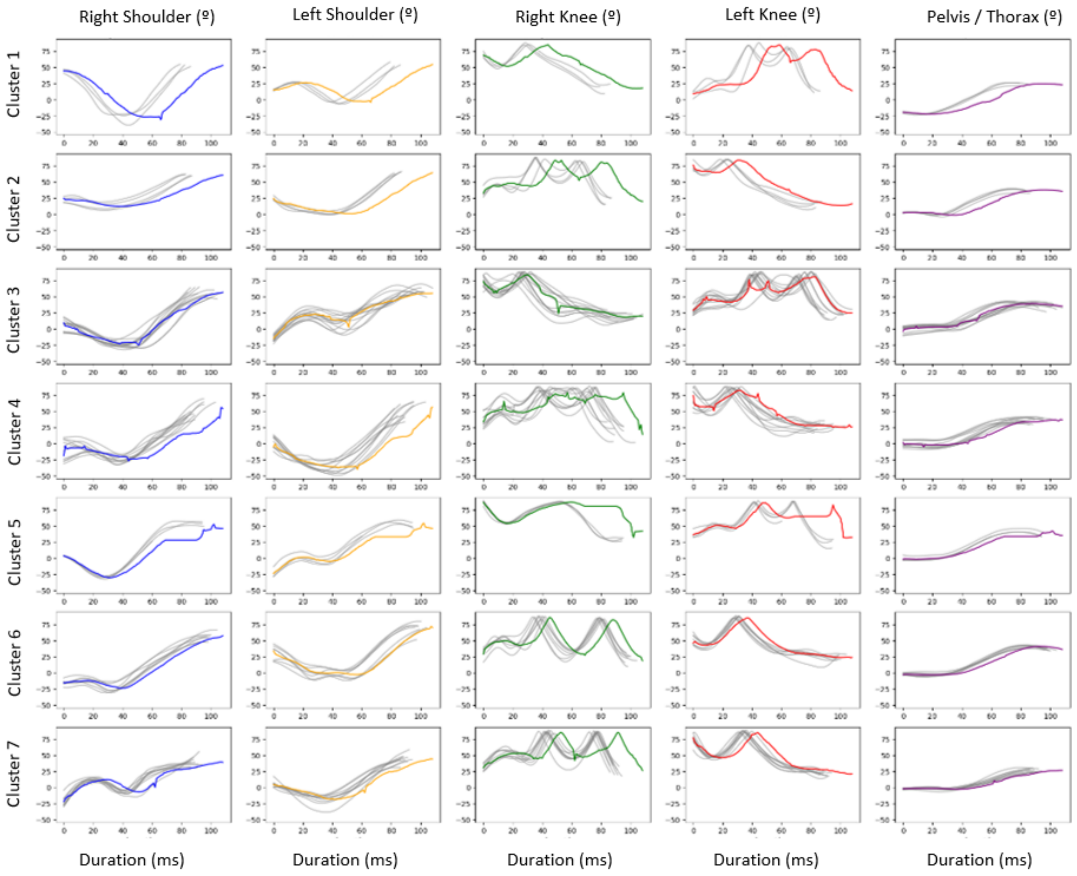

Figure S4. Cluster times series for phase 1 (i.e., from the last step on the carpet to the initial contact of both feet with the mini trampoline) considering the knees, shoulders and pelvis/thorax flexion/extension angles. The grey lines represent the time series of each trial.

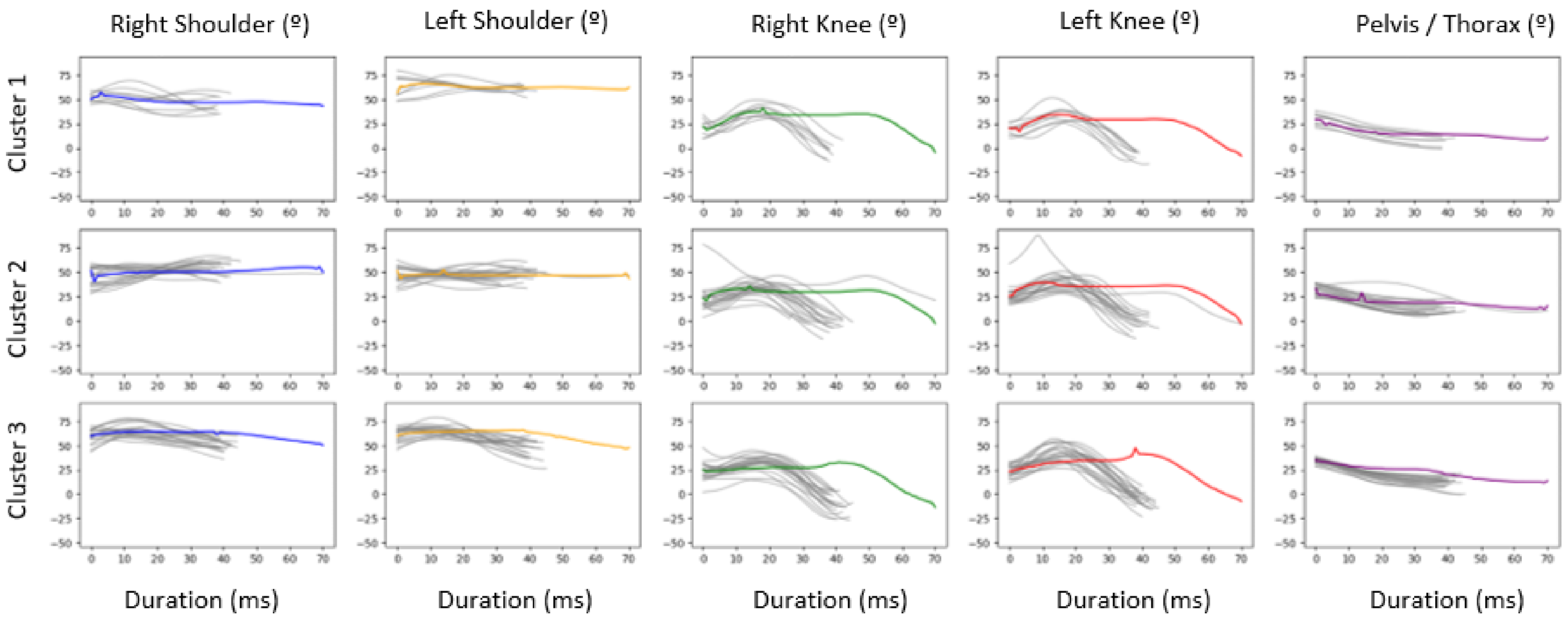

Figure S5. Cluster times series for phase 2 (i.e., from the initial contact to the take-off on the mini trampoline) considering the knees, shoulders and pelvis/thorax flexion/extension angles. The grey lines represent the time series of each trial.

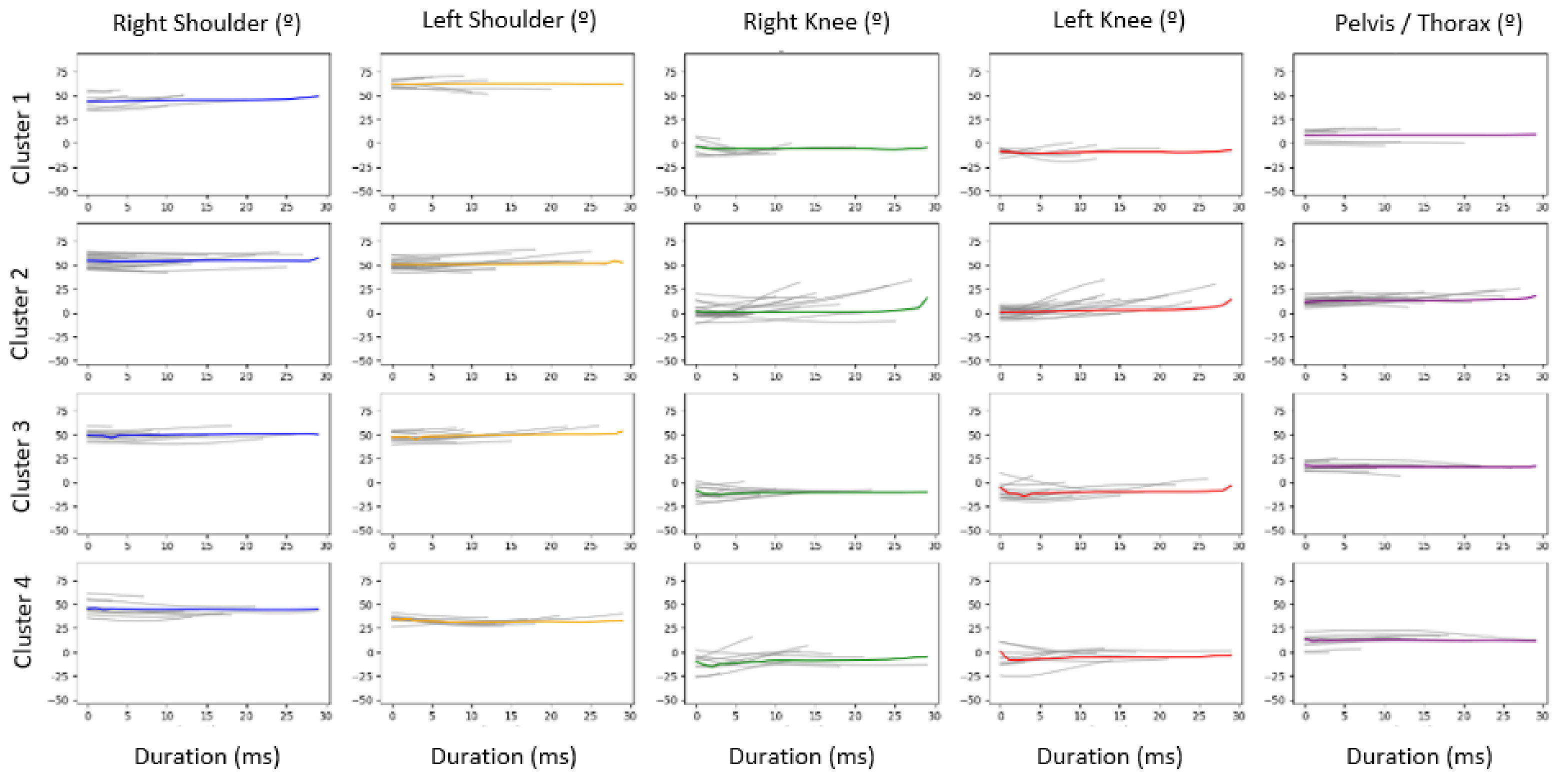

Figure S6. Cluster times series for phase 3 (i.e., from the take-off on the mini trampoline to the initial contact of both hands with the vaulting table) considering the knees, shoulders and pelvis/thorax flexion/extension angles. The grey lines represent the time series of each trial.

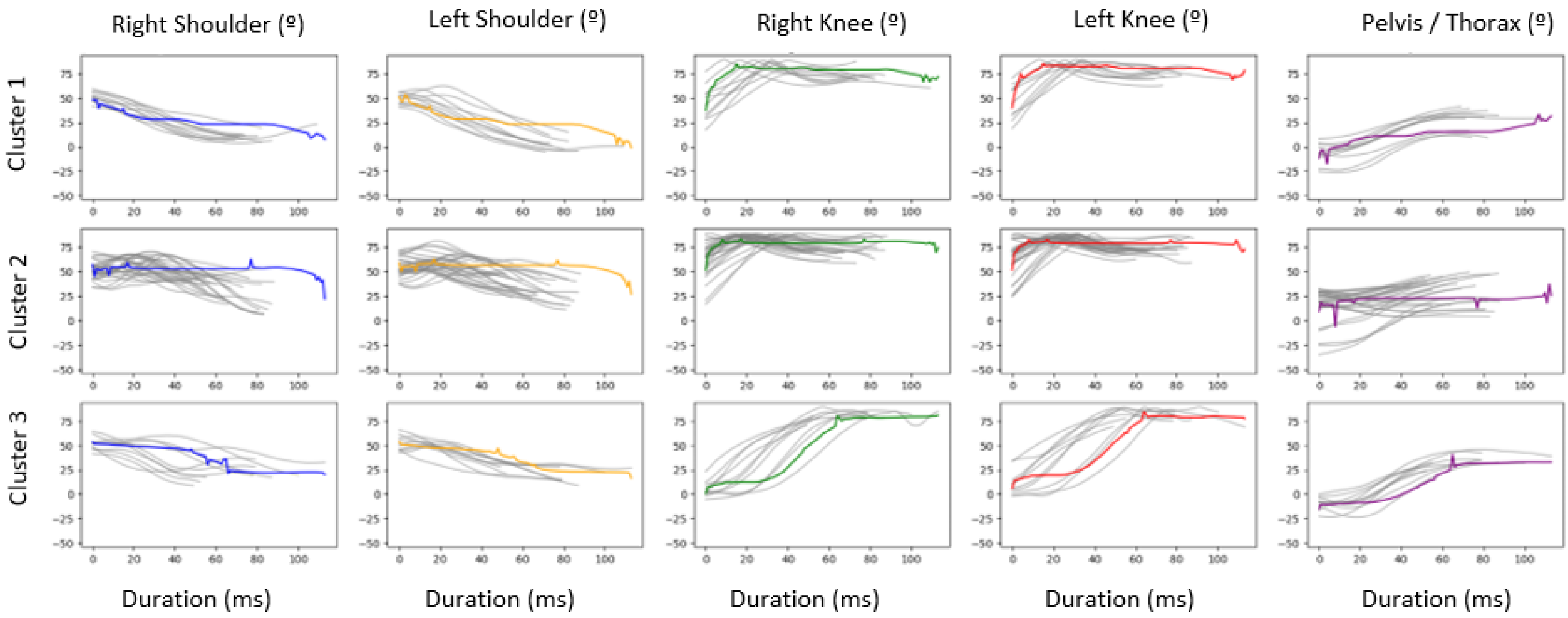

Figure S7. Cluster times series for phase 5 (i.e., from the take-off on the vaulting table to achieving the tucked body position during the aerial phase) considering the knees, shoulders and pelvis/thorax flexion/extension angles. The grey lines represent the time series of each trial.

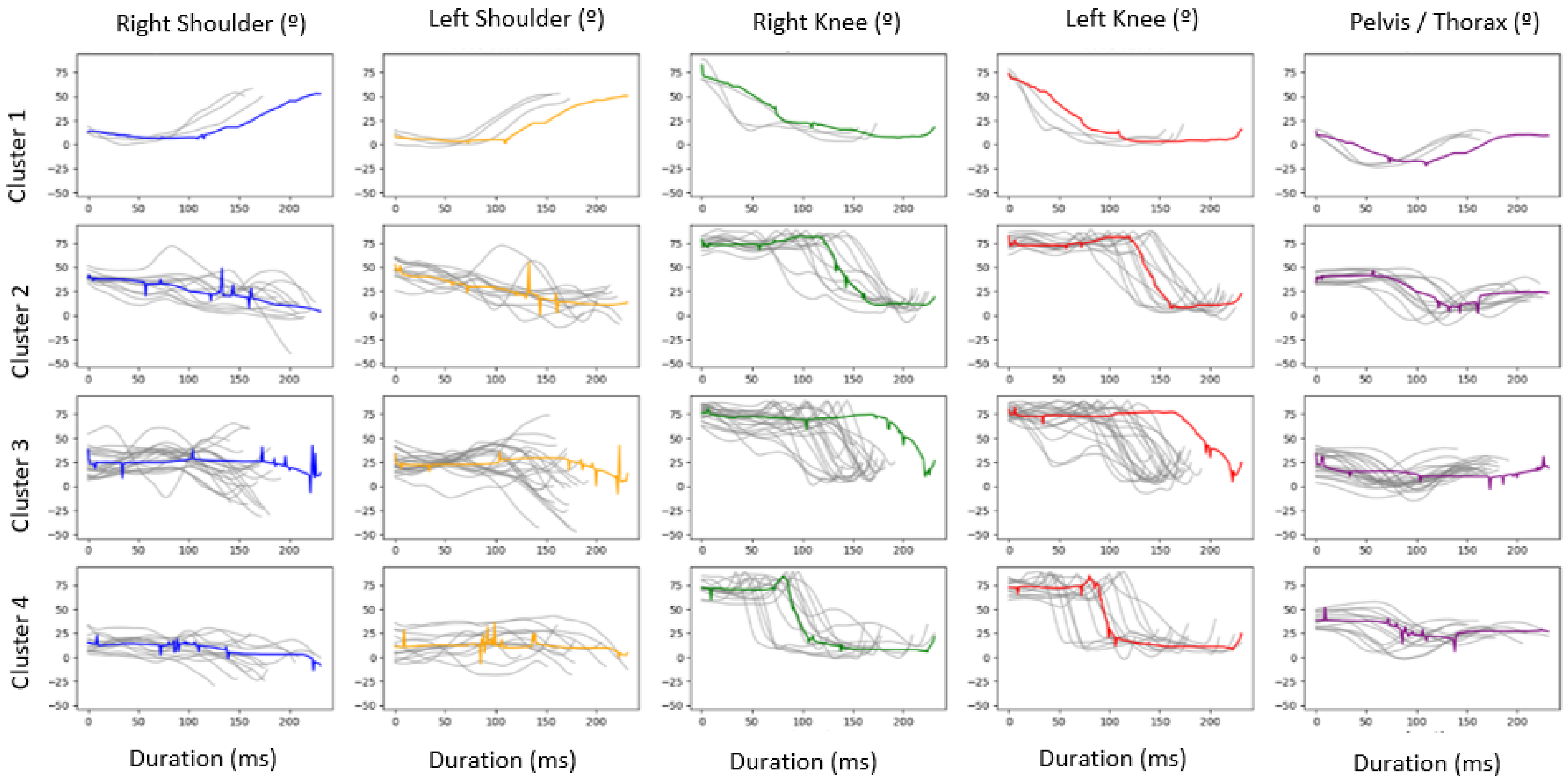

Figure S8. Cluster times series for phase 6 (i.e., from the tucked body position to landing with both feet on the landing mat) considering the knees, shoulders and pelvis/thorax flexion/extension angles. The grey lines represent the time series of each trial.
